# Identification and comprehensive analysis of MIPSs in Rosaceae and their expression under abiotic stresses in rose (*Rosa chinensis*)

**DOI:** 10.3389/fpls.2022.1021297

**Published:** 2022-11-03

**Authors:** Himanshi Gangwar, Priya Kumari, Vijay Gahlaut, Sanjay Kumar, Vandana Jaiswal

**Affiliations:** ^1^ Biotechnology Division, CSIR-Institute of Himalayan Bioresource Technology, Palampur, Himachal Pradesh, India; ^2^ Academy of Scientific and Innovative Research (AcSIR), Ghaziabad, India

**Keywords:** myo-inositol 1-phosphate synthase, phylogenetic analysis, gene structure, *cis*-elements, drought stress

## Abstract

The Myo-Inositol-1-phosphate synthase (MIPS) gene family is involved in the myo-inositol synthesis and plays a significant role in signal transduction, membrane biogenesis, oligosaccharides synthesis, auxin storage and transport, programmed cell death and abiotic stress tolerance in plants. This study comprehensively identified the MIPS genes in Rosaceae plant species, and 51 MIPS genes were identified from 26 Rosaceae species. The phylogenetic analysis divided the MIPSs into two clades (clade I; subfamily *Amygdaloideae* specific, and clade II; subfamily *Rosoideae* specific). MIPS genes of all 26 Rosaceae species consist of similar gene structure, motif and domain composition, which shows their conserved nature. The cis-regulatory elements (CREs) analysis revealed that most *Rosaceae* MIPS genes play a role in growth, development, and stress responses. Furthermore, the qRT-PCR analysis also revealed the involvement of *RcMIPS* gene in plant development and response to abiotic stresses, including drought and heat. The results of the present study contribute to the understanding of the biological function of Rosaceae MIPS genes, and that could be used in further functional validations.

## Introduction

The Myo-inositol phosphate synthase (MIPS) is a key enzyme in the myo-inositol biosynthesis pathway, that produces myo-inositol (Loewus and Loewus, 1980). Myo-inositol is an important component that regulates plant growth and development processes (Martin, 1998), membrane biogenesis (Roth, 2004), oligosaccharides synthesis (Karner et al., 2004), auxin storage and transport (Abreu and Aragao, 2007), programmed cell death (Meng et al., 2009), phytic acid biosynthesis (Ali et al., 2013), etc.

MIPS enzymes have been ubiquitously found in a wide range of organisms, including higher plants and animals, parasites, fungi, green algae, bacteria, and archaea, and it is thought to be a primordial protein/gene (Majumder et al., 1997). Recent reports have also specified its functional diversity and shared evolutionary clues from archaea to higher plants and animals (Majumder et al., 2003). A genome-wide investigation of MIPS copies in plant kingdom was carried out to unveil the mystery of sequence conservation and divergence (Hazra et al., 2019). MIPS evolved from a single common algal ancestor to seed plants, and functional diversification is perhaps controlled by genomic variations. The differences in the number and properties of MIPS genes result from tandem, block, whole genome duplication and subsequent divergence. Different MIPS gene variants acquired distinct functions during duplication and speciation events (Hazra et al., 2019).

Additionally, MIPSs are also involved in abiotic stress regulation in plants (Taji et al., 2006; Kaur et al., 2013; Tan et al., 2013; Sharma et al., 2020; Alok et al., 2022). For instance, under NaCl treatment, salt-resistant rice varieties (*Oryza sativa* L.) show increased activity of the chloroplast version of MIPS either at the seedling stage or on fully grown plants (Raychaudhuri and Majumder, 1996). The expression of wheat *TaMIPS2* was induced in various developmental seed stages during heat stress treatment. Unfertilized ovaries also showed greater *TaMIPS2* transcript levels, with considerable quantities remaining during the recovery phase, indicating that MIPS is important for heat stress recovery and flower development (Khurana et al., 2012). The expression of the *MfMIPS1* gene was induced by salt and drought stresses in *Medicago falcata*. The overexpressed *MfMIPS1* tobacco lines have increased levels of Myo-inositol, leading to enhanced tolerance to multiple abiotic stress, i.e., cold, salt and drought stress (Tan et al., 2013). The *IbMIPS1* overexpressed lines of sweet potato also exhibited increased salt and drought tolerance (Zhai et al., 2016). Recently, it was shown that *MdMIPS1* overexpressing apple lines showed enhanced drought stress tolerance (Hu et al., 2022). The above studies suggested that the *MIPS* genes were involved in the regulation of various abiotic stresses in plants.

The MIPS genes have been investigated thoroughly in several plant species, including the model plant Arabidopsis (Johnson and Sussex, 1995), maize (Larson and Raboy, 1999), sesame (Chun et al., 2003), rice (Feng and Yoshida, 2004), soybean (Kumari et al., 2012), wild rice; *Porteresia coarctata * (Basak et al., 2018), cotton (Ma et al., 2019), and oat (Qin et al., 2021). Compared with other species, studies on the characterization of MIPS genes and their function in growth and abiotic stress regulation in the *Rosaceae* family are limited. Therefore, in this study, we comprehensively analysed the distribution of MIPS genes in 26 different species of the *Rosaceae* family. We also characterized the MIPS gene with respect to its exon-intron structure, motif analysis, conserved domain, phylogenetic relationship, cis-regulatory elements, and gene ontology. Furthermore, the expression pattern of the rose *MIPS *gene was also performed in different tissues and response to the drought and heat stresses using qRT-PCR analysis. Altogether, this comprehensive information on MIPS genes of *Rosaceae* could be used for further functional validation and also provide insight into their functional role during abiotic stresses in roses.

## Materials and methods

### Sequence retrieval and identification of MIPSs in Rosaceae family

The genome sequences of 26 Rosaceae species available at the Genome Database for Rosaceae (GDR) (https://www.rosaceae.org; Jung et al., 2019) were utilized for the annotation and identification of the MIPS genes (more details were provided in **Table S1**). The three Arabidopsis MIPS protein sequences (IDs: AT2G22240, AT4G39800 and AT5G10170) were retrieved from the TAIR database (https://www.arabidopsis.org/) used as a query in the BLASTp program (e-value < 1× 10^-5^) to obtain MIPSs of the *Rosaceae*. Following that, all MIPS proteins were further verified for the presence of MIPS signature domains (NAD_5 Binding; PF07994 and Inos-1-P Synth; PF01658) using the Pfam database (http://pfam.xfam.org; El-Gebali et al., 2019). The molecular characteristics (aa length, molecular weight and isoelectric point) were predicted using the online tool ExPASy (http://web.expasy.org/; Artimo et al., 2012).

### Multiple sequence alignments and phylogenetic analysis of MIPSs

Using the MUSCLE package available in MEGA X software (Stecher et al., 2020), all 51 MIPS protein sequences representing 26 *Rosaceae* species were aligned. The phylogenetic tree was created using the maximum-likelihood (ML) method with Poisson model, pair-wise deletion and 1000 bootstraps in MEGA X software. The web programme iTOL (Letunic and Bork, 2007) has been used to visualize the phylogenetic tree.

### Gene structure and motif analysis of MIPSs

The exon-intron structure of the MIPS gene was determined using the online server GSDS 2.0 (Gene Structure and Display server 2.0; http://gsds.cbi.pku.edu.cn/Gsds_abou.php). The conserved motif was predicted by using the Motif Elicitation (MEME) program with the parameter set to find 20 motifs (http://meme-suit.org; Bailey et al., 2015) and visualize the structure using the TBtool software (Chen et al., 2020).

### *Cis*-regulatory elements analysis

To analyze the *cis* regulatory elements (CREs) of MIPSs, the upstream sequences (2,000 bp) of the start codon were fetched from the GDR database. The CREs were identified in MIPS genes using the PlantCARE online server (Lescot et al., 2002).

### Nuclear localization signals, subcellular localization and GO analysis

The NLS in MIPS were predicted using the web tool cNLS Mapper (Kosugi et al., 2009). Using the WoLF PSOPT II online resource (https://www.genscript.com/wolf-psort.html?src=leftbar), the subcellular localization of Rosaceae MIPSs was predicted. The rose MIPS gene ID were subjected to the ShinyGO v0.75 database (Ge et al., 2020) to obtain gene ontology (GO) annotation.

### Plant material and stress treatment

In this study, the rose genotype (*Rosa chinensis*) was used. The plants were grown in plastic pots containing soil, peat, and sand mixed in a ratio of 1:2:1 and under greenhouse conditions (photoperiod=14 h light/10 h dark and temperature=22 ± 2°C) for the expression analysis of the *RcMIPS* gene. For tissue-specific expression analysis, the following tissues, stem, leaf, bud and flower tissues were harvested at the flowering stage. To impose the heat stress (HS), plants were kept at 42°C for 24 h. For drought stress (DS) treatment, we irrigated the plants with 20% PEG 6000. Leaf samples were harvested at the following different times: 0h, 6h, 12h and 24h for both HS and DS. During the experiment, the control tissues were collected from the unstressed plants. In addition, we collected apple (*Malus domestica*) stem and leaf tissues for *MdMIPS* tissue-specific expression analyses. All of the tissues were collected in three biological replications and kept at -80°C till further use.

### RNA isolation and quantitative real-time PCR

The PureLinkTMRNA Mini Kit (Invitrogen) was used to isolate total RNA according to the manufacturer’s instructions. Then total RNA was converted to cDNA using Thermo Scientific’s Verso cDNA Synthesis Kit. In the QuantStudio 3 applied biosystems Real-Time PCR System, quantitative RT-PCR (qRT-PCR) was performed using SYBR green master mix (Applied Biosystems, Foster City, USA). ApE–A plasmid Editor (https://jorgensen.biology.utah.edu/wayned/ape/) was used to generate primers for the specified *RcMIPS*; Forward primer–AGGAGGAACACTTTGATTGGTGG and Reverse primer–GGTTTGTGGAGCTGAAAGGTTC. As an internal control, the housekeeping gene *RcActin* (RchiOBHm_Chr1g0322051) was employed. For expression of *MdMIPS*, Forward primer– ACAACTACGAGACCACCGAG and Reverse primer– GTGAGGGTTGAGCCATTGTT, as well as an internal control *nad5*, was used (Menzel et al., 2002). Each sample received three biological replicates and three technical replicates for qRT-PCR. The 2−ΔΔCt technique was used to determine the relative expression level of *RcMIPS* gene (Livak and Schmittgen, 2001).

## Results

### Distribution of MIPS genes in Rosaceae family

The genome-wide search through BLASTp searches and verification of the 26 Rosaceae species reference genomes identified a total of 51 MIPS genes (**Table 1**). These 26 species belong to two Rosaceae subfamilies (*Amygdaloideae* and *Rosoideae*). The genes were then named based on the reported genes in Arabidopsis and their chromosome location from top to bottom. The length of the *Rosaceae MIPS* gene ranged from 1042 (*MbMIPS3*) to 4079 bp (*FnMIPS*), and the average length was 2810 bp. The protein length ranged from 197aa (*MbMIPS2*) to 585 aa (*PpyMIPS2*), and the average length was 492 aa. The predicted molecular weight of Rosaceae MIPS protein varied from 22.0 kDa (*MdMIPS2*) to 64.6 kDa (*PpyMIPS2*), and the average molecular weight was 54.2 kDa. As shown in **Table 1**, there are variations in the number of MIPS genes among 26 *Rosaceae* species, it ranges from one (14 species see **Table 1**) to six (*Pyrus bretschneideri*). However, most of *Rosaceae* species (14 out of 26) were found to have single copy of MIPS gene and some have multiple copies like *Gillenia trifoliata* (2), *Malus domestica* (3), *Malus sieversii* (4), *Prunus domestica* (5), and *Pyrus bretschneideri* (6). To find out the reason behind the expansion of *Rosaceae* MIPS genes, we looked at gene duplication events in different *Rosaceae* genomes. A total of 43 pairs of duplications were found in the 26 *Rosaceae* species (for details see **Table S2**). We also calculated Ka/Ks value for duplicated gene pairs, the Ka/Ks values for all gene pairs were less than 1, with an average of 0.185 (**Table S2**). These results suggested that the *MIPS* genes in *Rosaceae* species are continuously evolving *via* purifying selection.

**Table 1:** Detailed information of *MIPS* genes from 26 Rosaceae plant species.

Subfamily/Plant species	Gene name	Gene ID	Chromosome; location*	Gene length (bp)	E	Protein		
						L	pI	MW(KDa)
**Amygdaloideae**								
*Gillenia trifoliata*	*GtMIPS1*	Gtrc1702g28691.t1	contig_1702_RaGOO; 37185	1625	8	200	8.15	22.21
	*GtMIPS2*	Gtr09g3310.t1	9; 4198065	2422	8	510	5.70	56.13
*Malus baccata*	*MbMIPS1*	MABA000009	scaffold1; 236392	3061	8	510	5.81	56.17
	*MbMIPS2*	MABA026953	scaffold531; 444372	3968	5	197	5.05	22.00
	*MbMIPS3*	MABA045782	scaffold7500; 1	1042	3	228	6.06	24.64
*Malus domestica*	*MdMIPS1*	Mdg_01B002770	1B; 9309046	2499	8	511	5.51	56.17
	*MdMIPS2*	Mdg_01A002870	1A; 10195884	2683	8	511	5.51	56.17
	*MdMIPS3*	Mdg_15A031080	15A; 36243538	2463	8	511	5.51	56.17
*Malus sieversii*	*MsiMIPS1*	Msi_01B002550	1B; 9466735	2500	8	511	5.51	56.17
	*MsiMIPS2*	Msi_01A002550	1A; 10121248	2498	8	511	5.51	56.17
	*MsiMIPS3*	Msi_15A029500	15A; 36274631	2463	8	511	5.70	56.16
	*MsiMIPS4*	Msi_15B030020	15B; 37382931	2463	8	511	5.81	56.17
*Malus sylvestris*	*MsyMIPS1*	Msy_01A002440	1A; 9097312	2491	8	511	5.51	56.17
	*MsyMIPS2*	Msy_01B002610	1B; 9839865	2493	8	511	5.51	56.20
	*MsyMIPS3*	Msy_15A028140	15A; 34739104	2466	8	511	5.70	56.20
*Prunus armeniaca*	*PaMIPS*	PARG01757m01	LG1; 13998443	3238	8	510	5.37	56.38
*Prunus avium*	*PavMIPS*	CpS0039G320m0	6; 15056760	3519	8	511	5.53	56.32
*Pyrus bretschneideri*	*PbMIPS1*	rna28649-v1.1-pbr	NA; 453960	2970	8	510	5.45	56.17
	*PbMIPS2*	rna33244-v1.1-pbr	1; 13180500	3671	8	510	5.51	56.17
	*PbMIPS3*	rna43611-v1.1-pbr	NA; 100556	2920	8	510	5.81	56.15
	*PbMIPS4*	rna43612-v1.1-pbr	NA; 248286	2975	8	510	5.81	56.18
	*PbMIPS5*	rna48894-v1.1-pbr	NA; 116663	2892	8	510	5.51	56.21
	*PbMIPS6*	rna52790-v1.1-pbr	NA; 16022	1647	1	547	6.45	60.72
*Pyrus communis*	*PcMIPS1*	pycom01g05310	1; 4948196	2506	8	511	5.61	56.25
	*PcMIPS2*	pycom15g29820	15; 26879374	1925	6	401	5.31	44.00
*Prunus domestica*	*PdMIPS1*	Pd.00g802200.m01-v1.0	scaffold17206; 51476	2901	8	510	5.61	56.36
	*PdMIPS2*	Pd.00g872390.m01-v1.0	scaffold5369; 2100778	2892	8	510	5.71	56.41
	*PdMIPS3*	Pd.00g936150.m01-v1.0	scaffold4694; 333516	2901	8	510	5.61	56.36
	*PdMIPS4*	Pd.00g945110.m01-v1.0	scaffold6401; 146743	2134	6	455	5.21	50.44
	*PdMIPS5*	Pd.00g1179490.m01-v1.0	scaffold2249; 1935754	2903	8	510	5.61	56.36
*Prunus dulcis*	*PduMIPS*	Prudul26A029919	6; 17553058	3386	8	511	5.38	56.43
*Prunus mandshurica*	*PmaMIPS*	Pruma.6G300700.t1.p1_1	6; 17234362	2892	8	511	5.31	56.33
*Prunus mira*	*PmMIPS*	Pmi06g1995	6; 19591497	3252	8	511	5.37	56.39
*Prunus persica*	*PpMIPS*	Prupe.6G180500.1	6; 18691855	3659	8	511	5.38	56.39
*Pyrus pyrifolia*	*PpyMIPS1*	Ppy01g0259.1	1; 6678562	2506	8	510	5.51	56.18
	*PpyMIPS2*	Ppy15g2730.1	15; 26879374	2728	9	585	7.70	64.61
*Prunus sibirica*	*PsiMIPS*	Prusib.6G327400.t1.p1_1	6; 19203084	3405	9	515	5.31	56.82
*Prunus sanyueli*	*PsMIPS*	PsSY0029765.1	LG04; 26042866	3288	8	511	5.61	56.36
*Pyrus ussuriensis x communis*	*PuMIPS1*	Pdr1g002830.1	1; 7456171	2918	8	511	5.51	56.21
	*PuMIPS2*	Pdr15g005650.1	15; 10111698	2950	8	511	5.70	56.18
**Rosoideae**								
*Fragaria ananassa*	*FaMIPS1*	FxaC_1g25440	1; 12191169	3327	8	511	5.38	56.48
	*FaMIPS2*	FxaC_2g28890	2; 14519335	3138	8	511	5.38	56.53
	*FaMIPS3*	FxaC_3g25020	3; 13883855	3145	8	511	5.38	56.47
*Fragaria nilgerrensis*	*FnMIPS*	evm.model.chr1.2310	1; 22143807	4079	8	511	5.38	56.47
*Fragaria viridis*	*FviMIPS*	evm.model.ctg108.72	1; 13382776	2701	8	511	5.31	56.45
*Fragaria vesca*	*FvMIPS1*	Fv2339_1g21380	1; 13374562	2909	8	510	5.38	56.47
	*FvMIPS2*	FvH4_1g21450	1; 13385153	3252	8	511	5.38	56.47
*Rubus chingii*	*RchMIPS*	LG01.1699	LG01; 14815667	2588	8	511	5.45	56.40
*Rosa chinensis*	*RcMIPS*	RC2G0276300	2; 27109903	2715	8	510	5.31	56.51
*Rubus occidentalis*	*RoMIPS*	Ro01_G10705	1; 11491881	2737	8	510	5.45	56.40
*Rosa rugosa*	*RrMIPS*	evm.model.Chr6.2780	6; 25125675	2626	3	510	5.31	56.47

*start position; E, Exons; L, length (aa); NA, not available.

### Phylogenetic analysis of Rosaceae MIPSs

**Figure 1:**
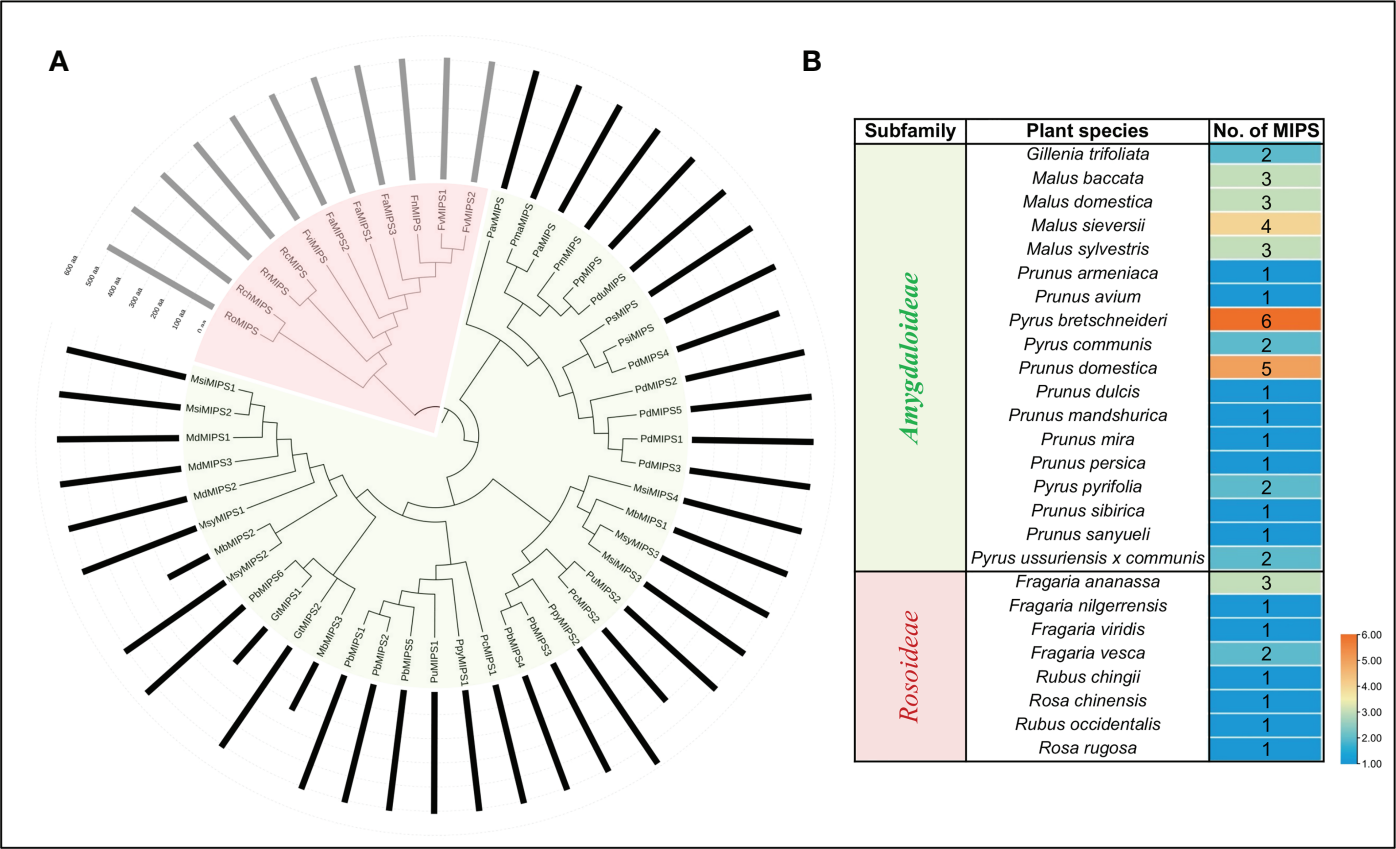
**(A)** Phylogenetic relationship among MIPSs belonging to the 26 Rosaceae plant species. **(B)** Table showing the number of MIPS genes present in each clade/sub-family.

To gain insights into the evolutionary relationship of the MIPSs in 26 *Rosaceae* species, a phylogenetic tree was made using ML-based multiple sequence alignments of 51 full-length protein sequences. The phylogenetic analysis divided the MIPSs into two clades (clade I and clade II) (**Figure 1**). Clade I had 40 MIPSs belonging to 18 species of *Rosaceae* subfamily *Amygdaloideae* and clade II had 11 MIPSs belonging to 8 species of *Rosaceae* subfamily *Rosoideae* (**Figure 1**).

### Gene structure, motif and domain composition of MIPSs

**Figure 2:**
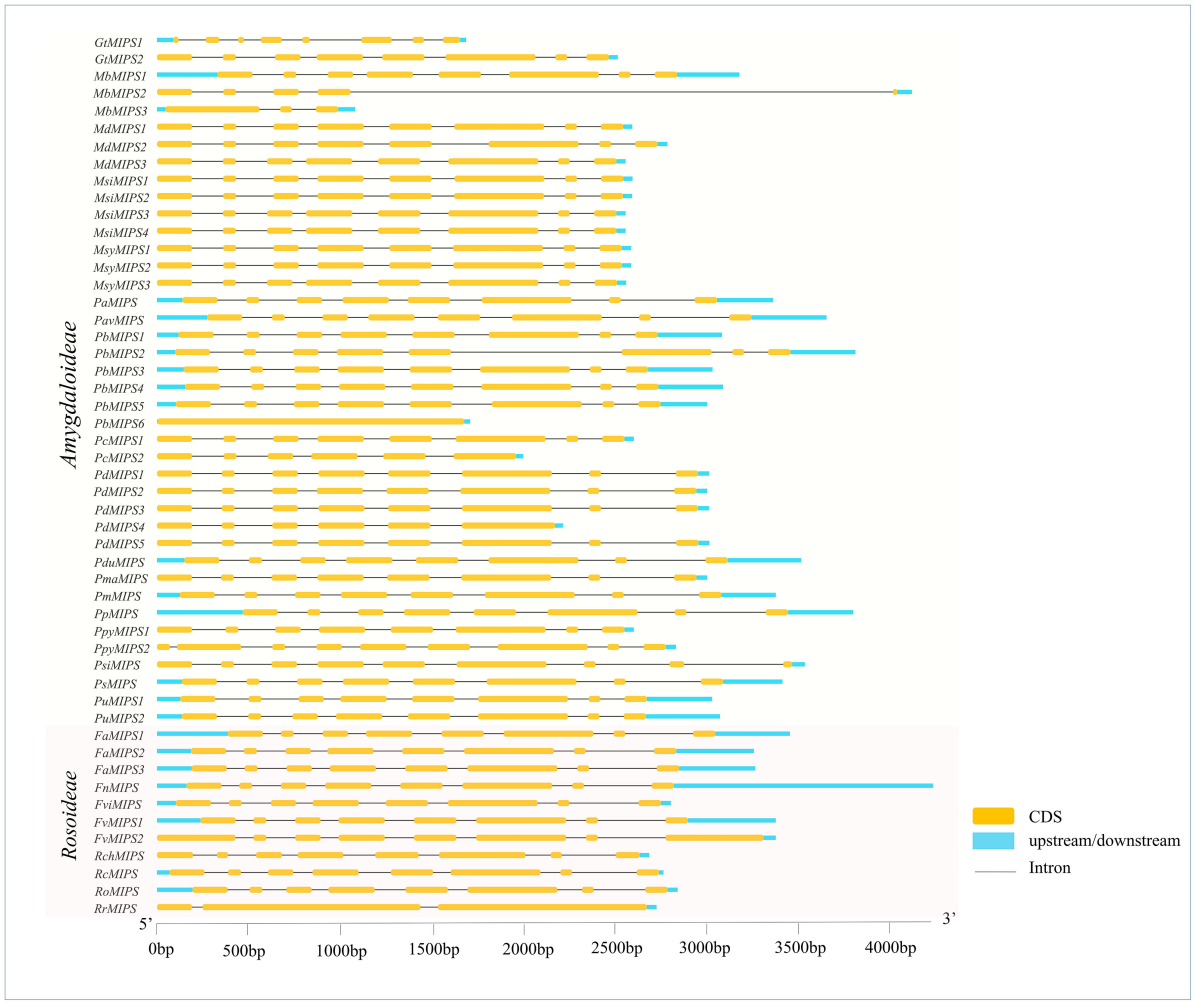
Exon–intron structures of Rosaceae MIPS genes. Exons are shown as yellow boxes, introns were denoted by thin dark grey lines and upstream/downstream regions are shown as sky-blue boxes. The lengths of exons and introns can be determined using the scale bar on the bottom.

**Figure 3:**
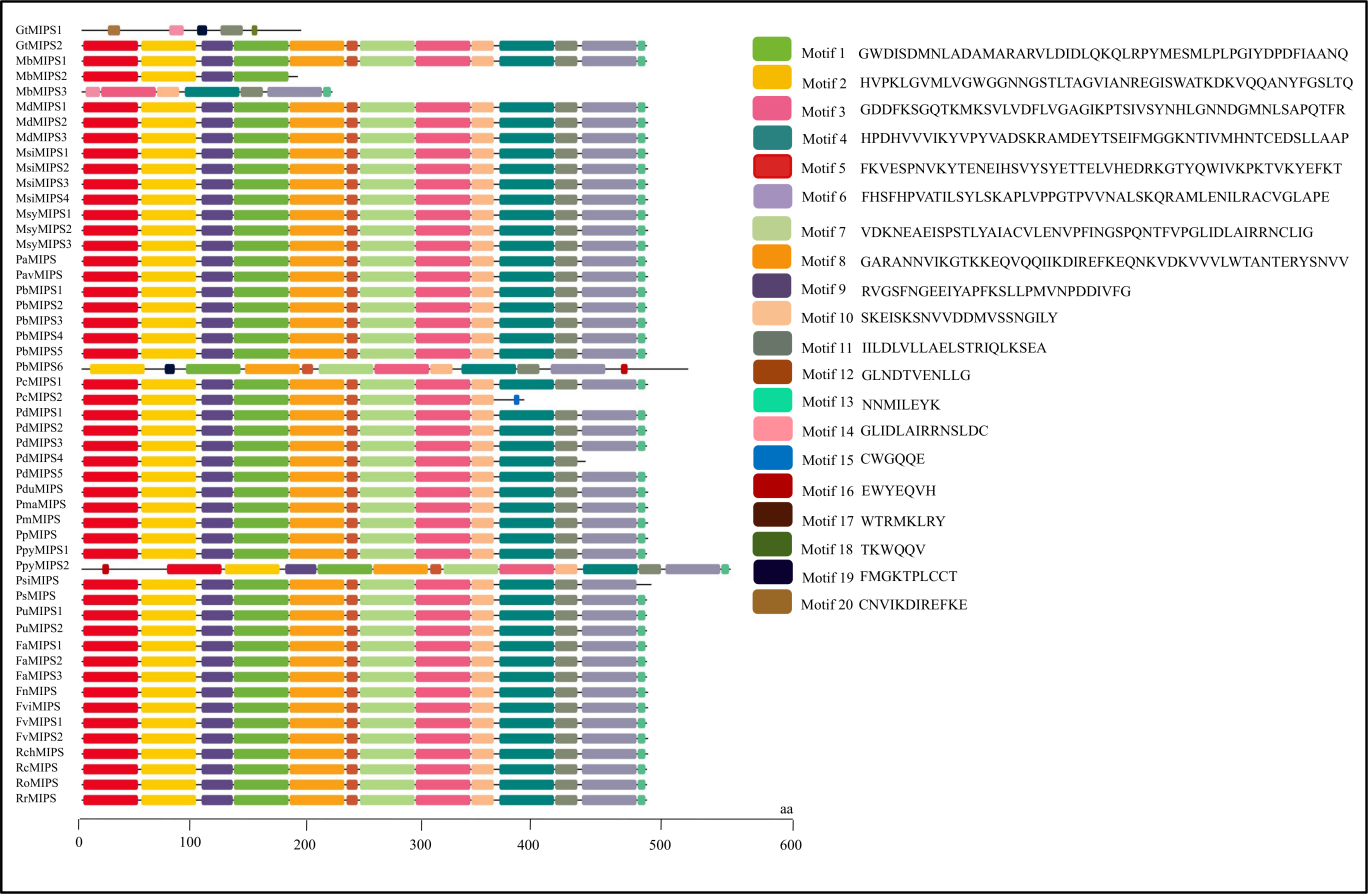
Motifs organization of Rosaceae MIPS proteins. Colour boxes represent the position of different motifs and box sizes show the length of motifs. Amino acid sequences for 20 different motifs (motif 1 to 20) are also provided on the right side.

**Figure 4:**
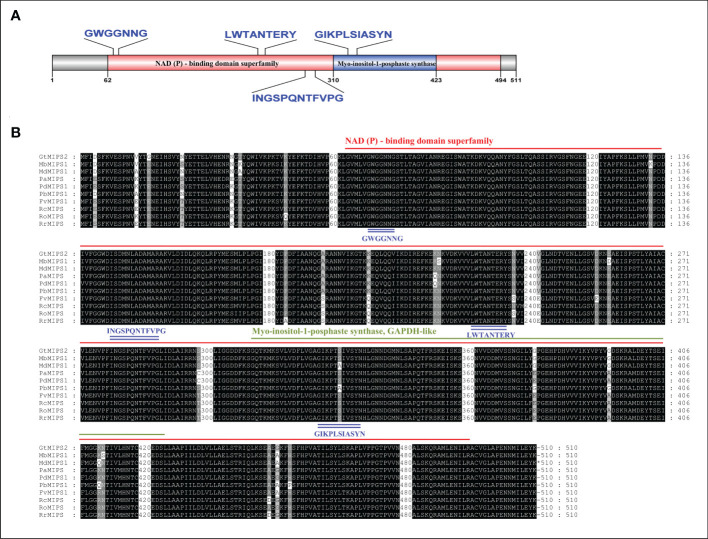
**(A)** Schematic representation of the conserved domain of Rosaceae MIPS protein. Two different conserved domains i.e., NAD binding domain (NAD_5_Binding) and myo-inositol-1-phosphate synthase (Inos-1-P_synth) are shown in different coloured boxes. **(B)** Multiple sequence alignment of ten representative Rosaceae MIPS proteins. Conserved domains are indicated with red and green underlines, the four stretches of conserved amino acid residues within domains are indicated by blue underlines.

The analysis of the exon-intron structures of the 51 MIPS genes in 26 *Rosaceae* species showed that the lengths of the MIPS genes were mainly distributed within the range of 1.5–4.0 kb. The number of exons ranges from one (*PbMIPS6*) to nine (*PsiMIPS* and *PpyMIPS2*). A total number of eight exons are most abundant and found in 43 *MIPS* genes (84%) (**Figure 2**). Motif distribution analysis predicted 20 different conserved motifs in 51 Rosaceae MIPS genes. The motif’s amino acid length was varied from 6 to 50. The 13 motifs (motifs 1 to 13) were present in all Rosaceae MIPS genes (except *GtMIPS1, MbMIPs2, MbMIPS3, PbMIPS6* and *PpyMIPS2*), and the organization pattern of these 13 motifs in the MIPS protein sequence was similar in all 26 Rosaceae plant species (**Figure 3**). These data suggest that MIPS is a highly conserved *Rosaceae* species. The conservation of MIPS genes was further confirmed as all Rosaceae plant species MIPS protein possessed NAD binding domain (NAD_5_Binding; PF07994) and myo-inositol-1-phosphate synthase (Inos-1-P_synth; PF01658) domain (**Figure 4A**). Additionally, the stretches of conserved amino acid residues such as (1) GWGGNNG, (2) LWTANTERY, (3) INGSPQNTFVPG, and (3) GIKPLSIASYN were also present in Rosaceae plant species (**Figure 4B**).

### Prediction of NLS and subcellular localization of MIPSs

The NLS prediction results showed that all the Rosaceae MIPSs had bipartite NLSs. The 51 *Rosaceae* MIPSs sequences have 41 NLSs sites (**Supplementary Table S3**). The PpyMIPS2 protein sequence also contains two monopartite sites in addition to bipartite NLSs. The results of subcellular localization prediction showed that all 51 Rosaceae MIPSs were localized in the cytoplasm (**Supplementary Table S3**).

### *Cis*−regulatory elements analysis of MIPSs

**Figure 5:**
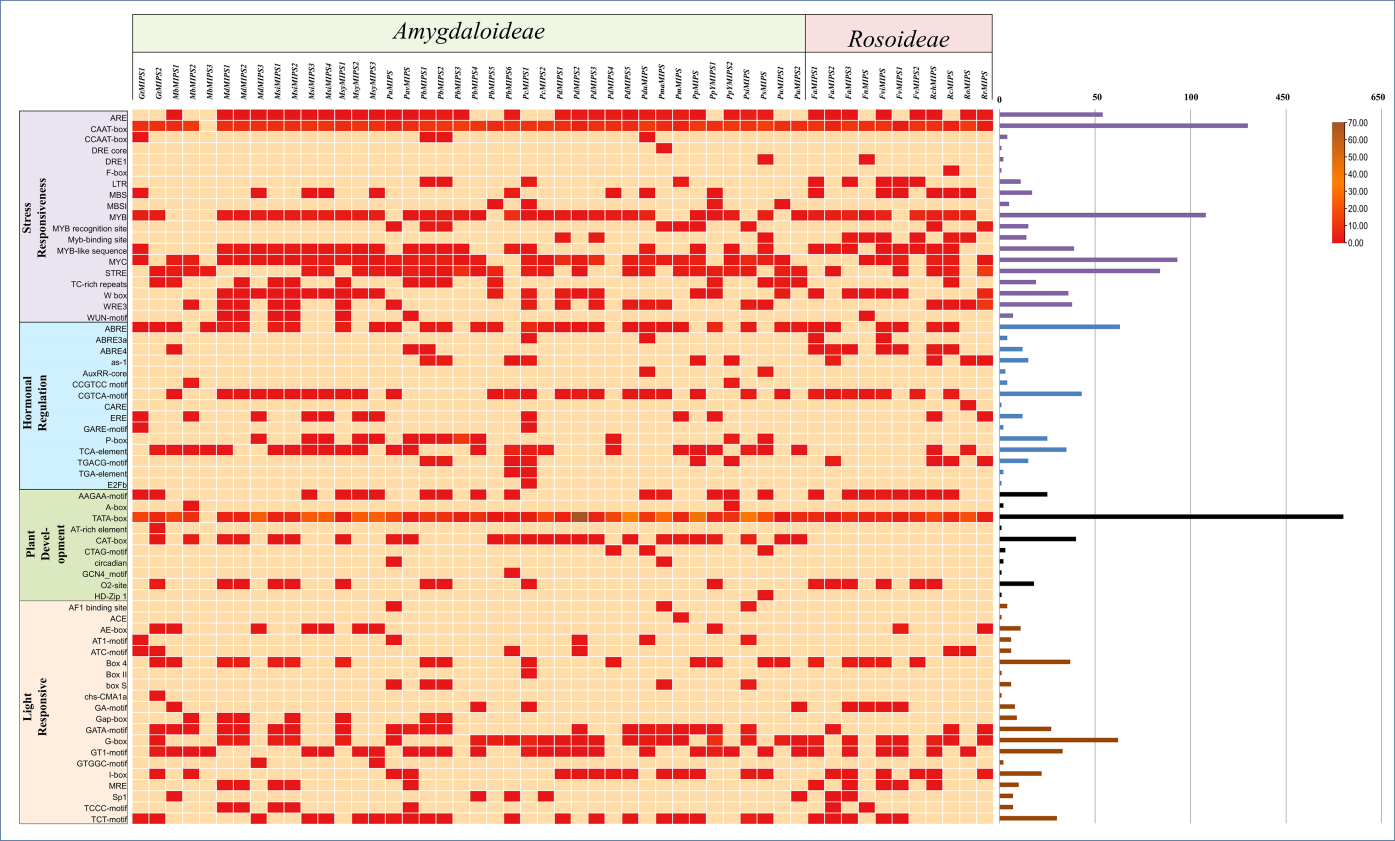
Showing the number of *cis*-regulatory elements (CREs) belonging to the following four categories (stress-responsive, hormonal regulation, plant development and light-responsive) per Rosaceae MIPS gene as a heatmap. Bar plots on the right represent the number of CREs for each MIPS gene.

To understand more about the possible role of *MIPS* genes in regulating growth and development and abiotic stress responses in Rosaceae species, we search for possible CREs in the two kb promoter regions of Rosaceae MIPSs genes. In total, 2243 representing 64 types of CREs were present in 51 Rosaceae MIPS genes (**Figure 5**). The preponderance of the cis-acting elements found were hormone response factors. The majority of the identified CREs were abiotic stress response elements (992; 44.22%), followed by plant growth-related elements (728; 32.45%), light-responsive elements (288; 12.83%), and hormone response factors (235; 10.47%).

Among the CREs of the stress response, drought-responsive CREs (ARE, DRE, MYB, MYC, MBS, F-box and ABRE) were predominantly present in the *Rosaceae* MIPS gene as compared to other stress-responsive elements. These findings suggest that *Rosaceae* MIPSs are involved in response to abiotic stresses, particularly drought. The 10 CREs involved in the growth and development of plants were identified in *Rosaceae* MIPS genes. These include AAGAA-motif, A-Box, TATA-box, AT-rich element, CAT-box, CTAG -motif, circadian, GCN4 motif, O_2_ site and HD-Zip1. The TATA-box and O2site CREs seemed to be the most prevalent and were detected in almost all MIPS genes. We also notice that three AT-rich element, CAT-box and CTAG -motif CREs found only in the *Amygdaloideae* subfamily of Rosaceae and absent in the *Rosoideae* subfamily. The 20 different types of light-responsive CREs were also predicted in Rosaceae MIPS genes. Among them, four CREs (Box 4, GATA-motif, GT1-motif and TCT-motif) seemed to be the most prevalent and appeared in almost all MIPS genes. Plant hormones-related CREs include the abscisic acid-responsive elements (ABRE), methyl jasmonate responsive elements (CGTCA-motif, TGACG-motif), salicylic acid-responsive elements (TCA-element), gibberellins responsive elements (P-box, GARE-motif), ethylene-responsive elements (ERE), and auxin-responsive elements (AuxRR-core, TGA-element). Altogether, these results indicate that MIPS genes may be involved in a variety of abiotic stress responses as well as plant growth regulation.

### GO annotation of MIPS gene

**Figure 6:**
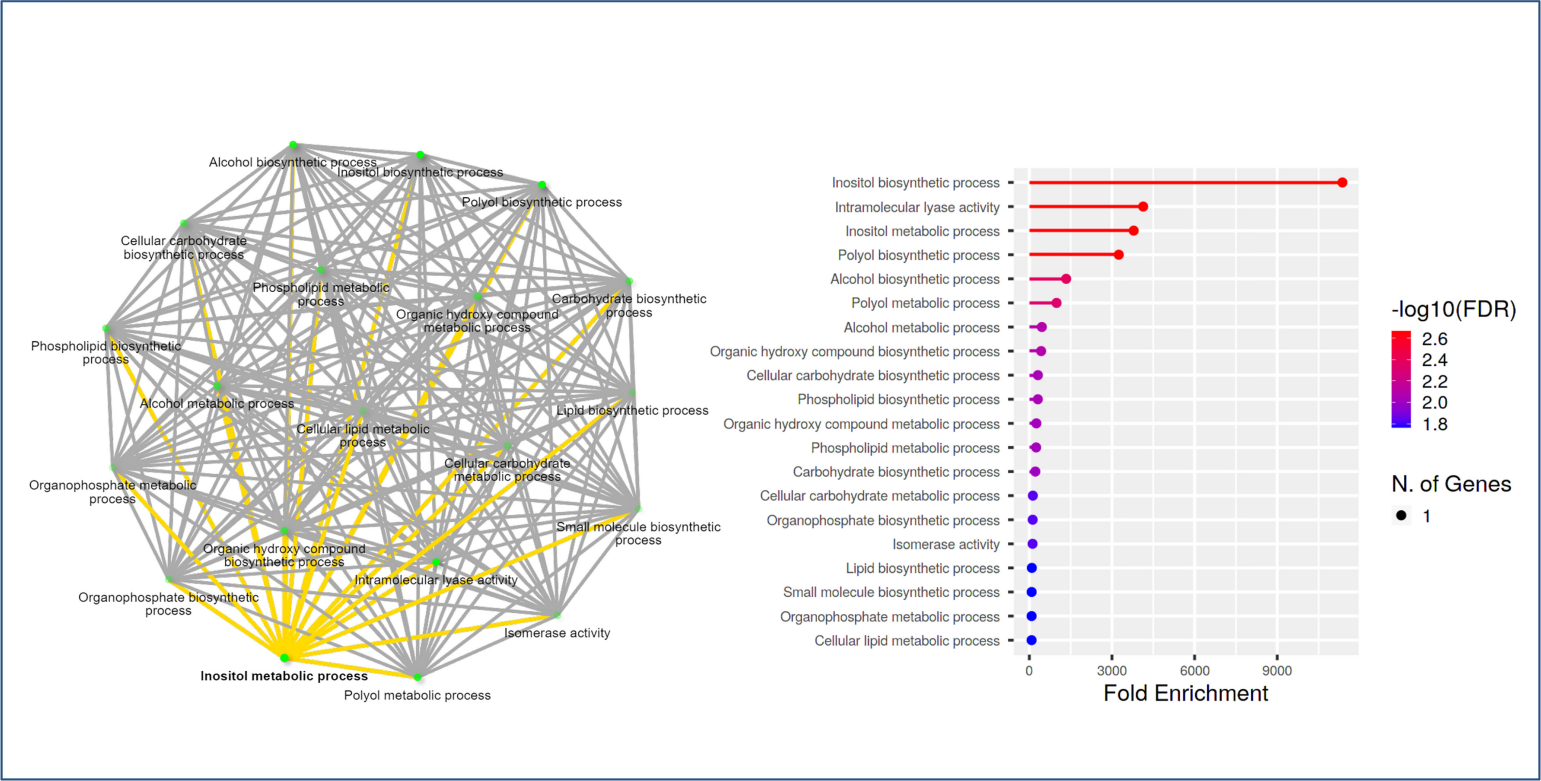
GO analysis using ShinyGo identified enriched biological processes of *RcMIPS* gene. The green dots represent the nodes for each GO biological process, while the lines (yellow and grey) represent the interaction between the nodes (minimum of 20% genes common between two connected) GO processes.

To gain more insight into the function of the Rosaceae *MIPS* genes, we also performed a GO enrichment analysis for the *RcMIPS* gene. The results of GO enrichment analysis revealed that the *RcMIPS* gene was involved in the Inositol (phytic acid) biosynthesis, intramolecular lyase activity, Inositol (phytic acid) metabolic process and Polyol biosynthesis process (**Figure 6**). This GO annotation suggested that the *MIPS* genes might play an important role in phytic acid biosynthesis.

### Expression profile of MIPS gene

**Figure 7:**
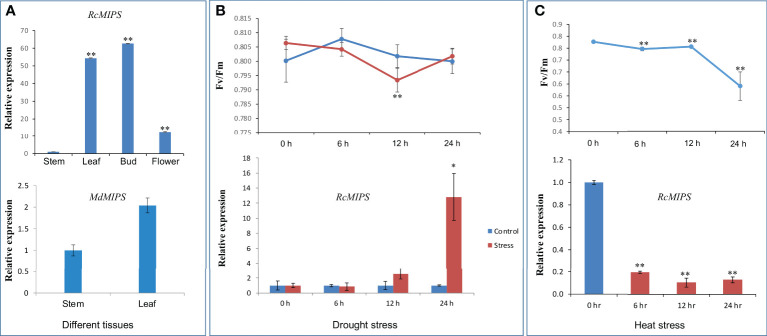
Relative expression level of MIPS in different plant tissue, during drought and heat stress, and effect of drought and heat stresses on quantum yield efficiency (Fv/Fm). **(A)** Relative expression level of *RcMIPS* and *MdMIPS* in different plant tissue. **(B)** Relative expression level of *RcMIPS* in leaf tissue at different times (0 h, 6 h, 12 h, and 24 h) treated with 20% PEG and effect of drought stress on quantum yield efficiency (Fv/Fm). **(C)** Relative expression level of *RcMIPS* in leaf tissue at different times (0 h, 6 h, 12 h, and 24 h) under heat stress at 42°C and effect of heat stress on quantum yield efficiency (Fv/Fm). * and ** represent the significant difference at *p <*0.05 and *p <*0.01, respectively using Student’s *t* test.

To investigate the putative biological role of Rosaceae MIPS genes, we performed expression analysis in *Malus domestica* (subfamily, *Amygdaloideae*) and *Rosa chinensis* (subfamily, *Rosoideae*). In *Malus domestica*, the MIPS gene was expressed in the leaf and stem. The expression level of the MIPS gene in both tissues was almost similar; only a slightly higher expression (although non-significant) was observed in the leaf as compared to stem tissue (**Figure 7A**). In case of rose, a significant difference in the relative expression of *RcMIPS* gene was observed in different plant tissue (**Figure 7A**). Among the four tissues, least expression was observed in the stem, however, the flower bud showed maximum expression of *RcMIPS* (62.66 fold higher than the stem). In the leaf and flower tissues, *RcMIPS* expressed 54.39 fold and 12.58 fold higher than the stem, respectively.

The significant difference of MIPS expression was observed in the case of rose; thus, further experiments were focused only in the rose. We performed drought stress treatment for six to 24 h on *R. chinensis* plants using 20% PEG-6000, and then we checked the expression of *RcMIPS* gene using qRT-PCR analysis. Expression analysis identified that under drought stress, *RcMIPS* gene was significantly upregulated (12.82-fold) as compared to control at only 24 h and before that its expression under drought stress was at par with control (**Figure 7B**). In the same tissues, we also calculated Fv/Fm to determine the physiological stress. As expected, at 0 h, no significant difference was observed in Fv/Fm values of control and drought stress tissue. Physiological stress was induced at 6 h and enhanced till 12 h, however, at 24 h physiological stress disappeared under drought stress. At 24 h, *RcMIPS* got upregulated and provided tolerance to plants to cope with stress conditions, and this might be one of the reasons that at 24 h no physiological stress was observed and Fv/Fm values of control tissue and drought stress tissue were almost same (**Figure 7B**). These results indicate that MIPS is possibly involved in drought stress tolerance in rose and a similar phenomenon might be operational in entire Rosaceae plant species.

We also explored the expression dynamics of *RcMIPS* under heat stress. For this purpose, plants were exposed to 42°C and leaf samples were collected at 0 h (control) and three-time points under heat stress i.e., 6 h, 12 h and 24 h. Under heat stress conditions, *RcMIPS* got significantly downregulated (**Figure 7C**). Further, physiological stress was also prominent in plants as Fv/Fm values significantly dropped under heat stress (**Figure 7C**).

## Discussion

The MIPS is a key enzyme in the myo-inositol biosynthesis pathway and has been involved in multiple biological roles in plants i.e., signal transduction, membrane biogenesis, oligosaccharides synthesis, auxin storage and transport, programmed cell death, phytic acid biosynthesis (Martin, 1998; Roth, 2004; Karner et al., 2004; Abreu and Aragao, 2007; Meng et al., 2009; Ali et al., 2013; Hazra et al., 2019). MIPS genes have been reported in various organisms including, bacteria, fungi, animals, and plants (Abid et al., 2009). However, no systematic identification and characterization of *MIPS* genes in the *Rosaceae* family have been reported. Here, by utilizing the reference genome sequence data of 26 *Rosaceae* species, we have identified 51 *MIPS* genes in Rosaceae (**Table 1**). The gene structures, phylogenetic relationships, conserved domain and motifs, CREs, GO annotation and expression profiling during drought and heat stresses for *RcMIPS* were also analyzed.

The number of MIPS genes among 26 Rosaceae species ranges from one to six and more than 50% of Rosaceae species had a single copy of MIPS genes. In earlier studies conducted in plants, it was observed that algal members, bryophyte (Marchantia), Gymnosperm (Ginkgo), Amborella, and important dicots (*Vitis vinifera*, *Eucalyptus grandis*, and *Daucus carota*) had only one copy of MIPS gene (Hegeman et al., 2001; Abid et al., 2009; Hazra et al., 2019). More than one copy of MIPS (two to four) was also reported in several plant species including rice (two MIPS copies) (Khurana et al., 2012), Arabidopsis (three MIPS copies) (Mitsuhashi et al., 2008), cotton and soybean (four MIPS copies) (Chappell et al., 2006; Ma et al., 2019). These results suggested that MIPSs are highly conserved and expanded and diversified in some higher plants including some *Rosaceae* species (*Gillenia trifoliata*, *Malus* spp.*, Pyrus* spp.*, Prunus* spp.*, Fragaria* spp.*) via* genome duplication and polyploidization events.

The phylogenetic analysis showed that 51 MIPSs belonging to 26 *Rosaceae* species were grouped into two distinct clades (*Amygdaloideae* and *Rosoideae* subfamily specific) (**Figure 1**). Interestingly, the MIPSs of 26 *Rosaceae* species were randomly dispersed in every clade, demonstrating that *Rosaceae* MIPSs were conserved and existed prior to *Rosaceae* plant species divergence. Further, most of the *Rosaceae* MIPS genes had similar exon-intron, motif and domain configurations (**Figures 2** & **3**). Earlier research has found that several key catalytic domains are highly conserved in MIPS amino acid sequence throughout evolutionary lines, indicating that they are catalytically active (Majumder et al., 2003; Hazra et al., 2019). The four highly conserved amino acid stretches i.e., GWGGNG, LWTANTERY, INGSPQNTFVPG and GIKPLSIASYN present in all 26 Rosaceae plant species (**Figure 4**) are also highly conserved in other plant species (Majumder et al., 2003; Hazra et al., 2019).

Most of the *Rosaceae* MIPS have NLS domains (**Table S3**), which are essential for these proteins’ precise targeting of the nucleus. The sub-cellular localization analysis predicts that *Rosaceae* MIPS were positioned in the cytoplasm (**Table S3**). As reported in earlier studies on different plant species MIPS is usually located in the cytoplasm but is also found in the nucleus, plasma membrane and endomembrane (Mitsuhashi et al., 2008; Donahue et al., 2010; Ray et al., 2010; Khurana et al., 2012; Ma et al., 2019). These results suggested that MIPS may be involved in signal transduction, membrane trafficking and regulation of gene expression.

We also predict 64 types CREs in the promoter of *Rosaceae* MIPS genes and these were associated with abiotic stress-response, plant development, light-responsive and hormonal regulation (**Figure 5**). In the promoter regions of MIPS genes in various plants, analogous CREs have been found (Khurana et al., 2012; Hazra et al., 2019; Ma et al., 2019). We also observed that drought-responsive CREs (ARE, DRE, MYB, MYC, MBS, F-box and ABRE) were predominantly present in the Rosaceae MIPS gene as compared to other stress-responsive elements. These findings suggest Rosaceae MIPS genes might play role in the growth and development, and stress response regulations in plants.

The MIPSs have been shown to be involved in the regulation of plant growth and development (Hazra et al., 2019). The significant difference (up to 62.66 fold) of *RcMIPS* in different plant parts like stem, leaf, bud, and flower suggested the important role of this gene in the growth and development of rose plants also (**Figure 7A**). MIPSs are also reported to play important role in abiotic stress tolerance including drought (Wei et al., 2010; Khurana et al., 2012; Kiranmai et al., 2018) and heat (Khurana et al., 2012). For instance, in groundnut, the *MIPS* gene was considered one of the drought-responsive genes and upregulated in transgenic lines which were more tolerant against drought as compared to wild type (Kiranmai et al., 2018). Similarly, in the case of other plant species like *Jatropha curcas* (Wang et al., 2011), *Ricinus communis* (Wei et al., 2010) *Ipomea batata* (Zhai et al., 2016) etc. *MIPS* genes were upregulated under drought stress. In the case of rice, splice variants of the *MIPS* gene (*OsMIPS1*) were also upregulated under drought stress (Khurana et al., 2012). Overexpression of chickpea *CaMIPS2* gene in Arabidopsis transgenic plants shows improved tolerance to drought stress (Kaur et al., 2013). Ectopic expression of *Medicago falcate* MIPS (*MfMIPS1*) gene in tobacco enhanced resistance to drought stress (Tan et al., 2013). During the present study, significant enhancement in expression of *MIPS* gene was observed under drought stress in the case of rose and suggested the positive role of the *MIPS* gene in providing tolerance against drought case of rose (**Figure 7B**). Additionally, we also identified drought-responsive elements CREs (i.e., ABRE, DRE, MYB and MYC) in the promoter sequence of *MIPS* genes which further suggested the involvement of MIPS in providing tolerance to plants under limited water conditions. Perhaps a similar kind of function may also be expected in the entire Rosaceae family. Further, biochemical studies also found an increase in myo-inositol levels under drought stress in the case of *Vigna umbellata* (Wanek and Richter, 1997) and *Cicer arietinum* (Boominathan et al., 2004). Altogether, MIPS genes were found to be a positive regulator which got upregulated under drought stress and increase the synthesis of Myo-inositol and ultimately provide tolerance to plants under drought stress.

Under heat stress, the expression of MIPS was reported to be different in different tissues. In the case of wheat, *TaMIPS* got upregulated in root and shoot tissues, however, downregulated in flower tissues under heat stress (Khurana et al., 2012). During the present study, *RcMIPS* got downregulated under heat stress and consequently, physiological stress (in form of Fv/Fm) was also observed (**Figure 7C**). These results may be explained by the fact that the genotype used in the present study was *R. chinensis*, and *R. chinensis* is a heat-susceptible species (Qi et al., 2021). Further, in *R. chinensis*, RNA seq study also suggested that genes involved in carbohydrate biosynthesis and cell wall biosynthesis genes were downregulated and resulted into physiological stress in *R. chinensis* (Li et al., 2019). All these observations suggested that allele of the *RcMIPS* gene in *R. chinensis* might be a susceptible allele for heat stress which got downregulated under heat stress and resulted in physiological stress in plants. However, the situation might be reversible with heat tolerant rose accession where MIPS genes (with tolerant allele) perhaps provide tolerance to plants under heat stress.

In conclusion, we identified 51 MIPS genes in 26 *Rosaceae* species of plants which were grouped into two clades. Gene structure analysis suggested one to nine exons in MIPS and the total gene length of MIPS were found up to 4079 bp. In promoter sequence, the presence of different elements including abiotic stress-responsive elements suggested the important role of MIPSs in different plant developmental processes as well as abiotic stresses. Expression analysis of *RcMIPS* empathized the potential role of MIPS in plant development, drought and heat stress tolerance. Altogether, the present study provides compressive preliminary information on *MIPS* genes which can be a good foundation for functional characterization of this important gene family.

## Data availability statement

The datasets presented in this study can be found in online repositories. The names of the repository/repositories and accession number(s) can be found in the article/**Supplementary Material**.

## Author contributions

HG and PK: Performed experiments, analysed data and wrote the first draft. VG and VJ: Conceptualization, supervision, writing and editing. SK: Overall supervision and funding acquisition. All authors contributed to the article and approved the submitted version.

## Funding

This work was supported by the CSIR, India under Grant MLP201; the Department of Science and Technology (DST) for the INSPIRE faculty award; and Science and Engineering Research Board (SERB) for the Early Career Research Award.

## Acknowledgments

This is a short text to acknowledge the contributions of specific colleagues, institutions, or agencies that aided the efforts of the authors. This study was supported by the Council of Scientific and Industrial Research (CSIR) for providing funds (MLP-201), VJ also thanks to the Science and Engineering Research Board (SERB) for the Early Career Research Award. VJ and VG thank the Department of Science and Technology (DST) for the INSPIRE faculty award. HG and PK thank CSIR for Junior Research Fellowship. This manuscript represents CSIR-IHBT communication number 5181.

## Conflict of interest

The authors declare that the research was conducted in the absence of any commercial or financial relationships that could be construed as a potential conflict of interest.

## Publisher’s note

All claims expressed in this article are solely those of the authors and do not necessarily represent those of their affiliated organizations, or those of the publisher, the editors and the reviewers. Any product that may be evaluated in this article, or claim that may be made by its manufacturer, is not guaranteed or endorsed by the publisher.
