# Supplementary material for: Identification and comprehensive analysis of MIPSs in Rosaceae and their expression under abiotic stresses in rose (Rosa chinensis)
Source: Front Plant Sci. 2022 Nov 3;13:1021297. doi: 10.3389/fpls.2022.1021297 (PMC9669799; doi:10.3389/fpls.2022.1021297)
Supplement: Supplementary file 1 [file DataSheet_1.pdf]

Supplementary Material

Table S1. A list of 26 Rosaceae species with their common name, genome version and available link at the Genome Database for Rosaceae (GDR).

Species	Common name	Version	Link
Gillenia trifoliata	Bowman's root	v1.0	https://www.rosaceae.org/bio_data/10816123
Malus baccata	Siberian crabapple	v1.0	https://www.rosaceae.org/bio_data/577
Malus domestica	Apple	v1.0	https://www.rosaceae.org/bio_data/10816131
Malus sieversii	Crabapple	v1.0	https://www.rosaceae.org/bio_data/10816132
Malus sylvestris	European crab apple	v1.0	https://www.rosaceae.org/bio_data/10816134
Prunus armeniaca	Apricot	v1.0	https://www.rosaceae.org/bio_data/603
Prunus avium	Sweet cherry	v1.0	https://www.rosaceae.org/bio_data/8674832
Pyrus bretschneideri	Chinese white pear	v1.1	https://www.rosaceae.org/bio_data/570
Pyrus communis	European pear	v2.0	https://www.rosaceae.org/bio_data/545
Prunus domestica	Plum	v1.0.a1	https://www.rosaceae.org/bio_data/531
Prunus dulcis	Almond	v2.0	https://www.rosaceae.org/bio_data/518
Prunus mandshurica	Manchurian apricot	v1.0	https://www.rosaceae.org/bio_data/10024324
Prunus mira	Smoothpit peach	v2.0	https://www.rosaceae.org/bio_data/12080707
Prunus persica	Peach	v2.0	https://www.rosaceae.org/bio_data/571
Pyrus pyrifolia	Asian pear	v1.0	https://www.rosaceae.org/bio_data/9597119
Prunus sibirica	Siberian apricot	v1.0	https://www.rosaceae.org/bio_data/9955981
Prunus sanyueli		v1.0	https://www.rosaceae.org/bio_data/11745274
Pyrus ussuriensis x communis	Harbin × European pear	v1.0	https://www.rosaceae.org/bio_data/608
Fragaria × ananassa	Garden Strawberry	v1.0.a2	https://www.rosaceae.org/bio_data/9642085
Fragaria nilgerrensis		v1.0	https://www.rosaceae.org/bio_data/12137894
Fragaria viridis	Green strawberry	v1.0	https://www.rosaceae.org/bio_data/9155217
Fragaria vesca	Woodland strawberry	v4.0.a2	https://www.rosaceae.org/bio_data/501
Rubus chingii	Chinese rasperry	v1.0	https://www.rosaceae.org/bio_data/11326199
Rosa chinensis	Chinese rose	v1.0	https://www.rosaceae.org/bio_data/457
Rubus occidentalis	Black raspberry	v3.0	https://www.rosaceae.org/bio_data/567
Rosa rugosa	Japanese rose	v1.0	https://www.rosaceae.org/bio_data/11775539

Table S2. List of duplicated gene pairs identified in MIPS genes of different Rosaceae species. The Ka represents the number of non-synonymous substitutions per non-synonymous site while Ks is the number of synonymous substitution per synonymous site.

Species name	Duplicate gene 1	Duplicate gene 2	Blast$_p$ result		Ka	Ks	Ka/Ks
			% Identity	Query cover			
Fragaria ananassa	*FaMIPS1*	*FaMIPS3*	99.80	100	0.001	0.006	0.148
	FaMIPS1	*FaMIPS2*	99.22	100	0.003	0.026	0.130
	FaMIPS2	*FaMIPS3*	99.41	100	0.003	0.026	0.097
Gillenia trifoliata	*GtMIPS1*	*GtMIPS2*	59.63	99	0.309	0.451	0.686
Malus domestica	*MdMIPS1*	*MdMIPS3*	100	100	0.014	0.164	0.084
	MdMIPS1	*MdMIPS2*	100	100	0.000	0.000	NaN
	MdMIPS2	*MdMIPS3*	100	100	0.014	0.164	0.084
Malus sieversii	*MsiMIPS1*	*MsiMIPS2*	100	100	0.000	0.003	0.000
	MsiMIPS1	*MsiMIPS4*	97.07	100	0.014	0.164	0.084
	MsiMIPS1	*MsiMIPS3*	96.67	100	0.016	0.168	0.092
	MsiMIPS2	*MsiMIPS1*	100	100	0.000	0.003	0.000
	MsiMIPS2	*MsiMIPS4*	97.06	100	0.014	0.168	0.082
	MsiMIPS2	*MsiMIPS3*	96.67	100	0.016	0.171	0.091
	MsiMIPS3	*MsiMIPS4*	99.61	100	0.002	0.003	0.600
Malus sylvestris	*MsyMIPS1*	*MsyMIPS2*	99.61	100	0.002	0.006	0.300
	MsyMIPS1	*MsyMIPS3*	96.86	100	0.015	0.164	0.089
	MsyMIPS2	*MsyMIPS3*	96.48	100	0.016	0.157	0.104
Prunus domestica	*PdMIPS1*	*PdMIPS3*	100	100	0.000	0.000	NaN
	PdMIPS1	*PdMIPS5*	100	100	0.000	0.003	0.000
	PdMIPS1	*PdMIPS2*	99.80	100	0.001	0.003	0.299
	PdMIPS1	*PdMIPS4*	100	88	0.003	0.012	0.267
	PdMIPS2	*PdMIPS3*	99.80	100	0.001	0.003	0.299
	PdMIPS2	*PdMIPS5*	99.80	100	0.001	0.000	-
	PdMIPS2	*PdMIPS4*	99.78	88	0.004	0.009	0.479
	PdMIPS3	*PdMIPS5*	100	100	0.000	0.003	0.000
	PdMIPS3	*PdMIPS4*	100	88	0.003	0.012	0.267
	PdMIPS4	*PdMIPS5*	100	98	0.003	0.009	0.369
Pyrus bretschneideri	*PbMIPS1*	*PbMIPS5*	99.80	100	0.001	0.006	0.150
	PbMIPS1	*PbMIPS2*	99.61	100	0.002	0.006	0.301
	PbMIPS1	*PbMIPS4*	96.67	100	0.015	0.185	0.079
	PbMIPS1	*PbMIPS3*	96.47	100	0.016	0.185	0.084
	PbMIPS1	*PbMIPS6*	58.19	89	0.370	3.644	0.102
	PbMIPS2	*PbMIPS5*	99.80	100	0.001	0.000	-
	PbMIPS2	*PbMIPS3*	96.67	100	0.016	0.185	0.084
	PbMIPS2	*PbMIPS4*	96.67	100	0.015	0.185	0.079
	PbMIPS3	*PbMIPS4*	99.80	100	0.001	0.000	-
	PbMIPS3	*PbMIPS5*	96.67	100	0.015	0.185	0.079
	PbMIPS4	*PbMIPS5*	96.86	100	0.014	0.186	0.074
	PbMIPS4	*PbMIPS6*	58.40	89	0.373	2.939	0.127
	PbMIPS5	*PbMIPS6*	58.19	100	0.369	4.032	0.092
Pyrus communis	*PcMIPS1*	*PcMIPS2*	94.55	75	0.049	0.244	0.199
Pyrus pyrifolia	*PpyMIPS1*	*PpyMIPS2*	96.078	100	0.017	0.185	0.093
Pyrus ussuriensis x communis	*PuMIPS1*	*PuMIPS2*	96.471	100	0.016	0.182	0.085

Table S3. The details of nuclear localization signals (NLSs) and predicted subcellular localization of Rosaceae MIPS genes.

Gene	Nuclear localization signals (NLS)				Subcellular localization
	Position	Score	Sequence	Mono/ Bipartite	
GtMIPS1	-	-	-	-	Cytoplasm
GtMIPS2	193	4.9	RANNVIKGTKKEQLQQVIKDIREFKEKNKVDKV	Bi	Cytoplasm
MbMIPS1	193	5.0	RANNVIKGTKKEQLQQVIKDIREFKEKSKVDKV	Bi	Cytoplasm
MbMIPS2	-	-	-	-	Cytoplasm
MbMIPS3	-	-	-	-	Cytoplasm
MdMIPS1	-	-	-	-	Cytoplasm
MdMIPS2	-	-	-	-	Cytoplasm
MdMIPS3	-	-	-	-	Cytoplasm
MsiMIPS1	-	-	-	-	Cytoplasm
MsiMIPS2	-	-	-	-	Cytoplasm
MsiMIPS3	193	5.0	RANNVIKGTKKEQLQQVIKDIREFKEKSKVDKV	Bi	Cytoplasm
MsiMIPS4	193	5.0	RANNVIKGTKKEQLQQVIKDIREFKEKSKVDKV	Bi	Cytoplasm
MsyMIPS1	-	-	-	-	Cytoplasm
MsyMIPS2	-	-	-	-	Cytoplasm
MsyMIPS3	193	5.0	RANNVIKGTKKEQLQQVIKDIREFKEKSKVDKV	Bi	Cytoplasm
PaMIPS	193	4.7	RANNVIKGTKKEQVQQIIKDIREFKEQNKVDKV	Bi	Cytoplasm
PavMIPS	193	4.7	RANNVIKGTKKEQVQQIIKDIREFKEQNKVDKV	Bi	Cytoplasm
PdMIPS1	193	4.7	RANNVIKGTKKEQVQQIIKDIREFKEQNKVDKV	Bi	Cytoplasm
PdMIPS2	193	4.7	RANNVIKGTKKEQVQQIIKDIREFKEQNKVDKV	Bi	Cytoplasm
PdMIPS3	193	4.7	RANNVIKGTKKEQVQQIIKDIREFKEQNKVDKV	Bi	Cytoplasm
PdMIPS4	193	4.7	RANNVIKGTKKEQVQQIIKDIREFKEQNKVDKV	Bi	Cytoplasm
PdMIPS5	193	4.7	RANNVIKGTKKEQVQQIIKDIREFKEQNKVDKV	Bi	Cytoplasm
PduMIPS	193	5.1	RANNVIKGTKKEQVQQIIEDIREFKEQNKVDKV	Bi	Cytoplasm
PmaMIPS	193	4.7	RANNVIKGTKKEQVQQIIKDIREFKEQNKVDKV	Bi	Cytoplasm
PmMIPS	193	5.1	RANNVIKGTKKEQVQQIIEDIREFKEQNKVDKV	Bi	Cytoplasm
PpMIPS	193	5.1	RANNVIKGTKKEQVQQIIEDIREFKEQNKVDKV	Bi	Cytoplasm
PsMIPS	193	4.7	RANNVIKGTKKEQVQQIIKDIREFKEQNKVDKV	Bi	Cytoplasm
PsiMIPS	193	4.7	RANNVIKGTKKEQVQQIIKDIREFKEQNKVDKV	Bi	Cytoplasm
PbMIPS1	-	-	-	-	Cytoplasm
PbMIPS2	-	-	-	-	Cytoplasm
PbMIPS3	193	5.0	RANNVIKGTKKEQLQQVIKDIREFKEKSKVDKV	Bi	Cytoplasm
PbMIPS4	193	5.0	RANNVIKGTKKEQLQQVIKDIREFKEKSKVDKV	Bi	Cytoplasm
PbMIPS5	-	-	-	-	Cytoplasm
PbMIPS6	108	5.2	EAMKRAKVLDLNLQKQLYPMMEKIVPRKAL	Bi	Cytoplasm
PcMIPS1	-	-	-	-	Cytoplasm
PcMIPS2	193	5.4	RANNVIKGTKKEQLQQVIEDIREFKEKSKVDKV	Bi	Cytoplasm
PpyMIPS1	-	-	-	-	Cytoplasm
PpyMIPS2	268	5.0	RANNVIKGTKKEQLQQVIKDIREFKEKSKVDKV	Bi	Cytoplasm
	64	4.0	LGRKRRKKKTEKMF	Mono	
	10	10.0	LGRKRRKKKT	Mono	
PuMIPS1	-	-	-	-	Cytoplasm
PuMIPS2	193	5.4	RANNVIKGTKKEQLQQVIEDIREFKEKSKVDKV	Bi	Cytoplasm
FaMIPS1	193	5.8	RANNVIKGTKQEQVQQIIKDIREFKEKNKVDKV	Bi	Cytoplasm
FaMIPS2	193	5.1	RANNMIKGTKQEQVQQIIKDIREFKEKNKLDKV	Bi	Cytoplasm
FaMIPS3	193	4.8	RANNVIKGTKQEQVQQIIKDIREFKEKNKVDKV	Bi	Cytoplasm
FnMIPS	193	4.8	RANNVIKGTKQEQVQQIIKDIREFKEKNKVDKV	Bi	Cytoplasm
FviMIPS	-	-	-	-	Cytoplasm
FvMIPS1	193	4.8	RANNVIKGTKQEQVQQIIKDIREFKEKNKVDKV	Bi	Cytoplasm
FvMIPS2	193	4.8	RANNVIKGTKQEQVQQIIKDIREFKEKNKVDKV	Bi	Cytoplasm

RchMIPS	193	4.8	RANNVIKGTKQEQVQQIIKDIREFKEKNKVDKV	Bi	Cytoplasm
RcMIPS	193	4.8	RANNVIKGTKQEQVQQIIKDIREFKEKNKVDKV	Bi	Cytoplasm
RoMIPS	193	4.8	RANNVIKGTKQEQVQQIIKDIREFKEKNKVDKV	Bi	Cytoplasm
RrMIPS	193	4.8	RANNVIKGTKQEQVQQIIKDIREFKEKNKVDKV	Bi	Cytoplasm
